# Effect of Fluid Friction on Interstitial Fluid Flow Coupled with Blood Flow through Solid Tumor Microvascular Network

**DOI:** 10.1155/2015/673426

**Published:** 2015-04-19

**Authors:** Mostafa Sefidgar, M. Soltani, Kaamran Raahemifar, Hossein Bazmara

**Affiliations:** ^1^Department of Engineering and Technology, IKI University, Qazvin 34148-96818, Iran; ^2^Department of Mechanical Engineering, K. N. T. University of Technology, Tehran 19991-43344, Iran; ^3^Division of Nuclear Medicine, Department of Radiology and Radiological Science, Johns Hopkins University School of Medicine, Baltimore, MD 21218-2608, USA; ^4^Electrical & Computer Department, Ryerson University, Toronto, ON, Canada

## Abstract

A solid tumor is investigated as porous media for fluid flow simulation. Most of the studies use Darcy model for porous media. In Darcy model, the fluid friction is neglected and a few simplified assumptions are implemented. In this study, the effect of these assumptions is studied by considering Brinkman model. A multiscale mathematical method which calculates fluid flow to a solid tumor is used in this study to investigate how neglecting fluid friction affects the solid tumor simulation. The mathematical method involves processes such as blood flow through vessels and solute and fluid diffusion, convective transport in extracellular matrix, and extravasation from blood vessels. The sprouting angiogenesis model is used for generating capillary network and then fluid flow governing equations are implemented to calculate blood flow through the tumor-induced capillary network. Finally, the two models of porous media are used for modeling fluid flow in normal and tumor tissues in three different shapes of tumors. Simulations of interstitial fluid transport in a solid tumor demonstrate that the simplifications used in Darcy model affect the interstitial velocity and Brinkman model predicts a lower value for interstitial velocity than the values that Darcy model predicts.

## 1. Introduction

Based on findings from clinical applications, most drug treatments fail to eliminate malignant tumors completely even though drug delivery through systemic administration may inhibit their growth [1]. Therefore, better understanding of tumor formation is crucial in developing more effective therapeutics [2]. For this purpose, nowadays, solid tumor modeling and simulation results are used to predict how therapeutic drugs are transported to tumor cells by blood flow through capillaries and tissues.

There are two approaches in the simulation of fluid flow to solid tumors: macroscopic and microscopic. In the macroscopic approach, only the distribution of variables, such as interstitial pressure and concentration, over the length scale of the tumor radius is important. In the microscopic approach, characteristics such as structure of microvascular network, blood flow through microvascular network, and interaction between microvascular wall and flow in peripheral flow are involved directly in the model.

Baxter and Jain [3–5] by considering macroscopic view used a continuum porous media model to simulate interstitial flow and solute transport in solid tumors. Improving the 1D model of Baxter and Jain [3–5] and Saltzman and Radomsky [6] by considering the complex 3D geometry, Wang et al. [7–9] developed a simulation framework of drug delivery to tumors. Wang and Li [7] used modified MRI images for computational geometry. They considered interstitial fluid flow with blood and lymphatic drainage in their model. Wang et al. [8] studied the effect of elevated interstitial pressure, convective flux, and blood drainage on the delivery of specified solute to brain tumors.

Soltani and Chen [1] developed a mathematical model in the spherical tumors to introduce two new parameters: the critical tumor radius and critical necrotic radius. They also used their model to study the effects of tumor shape and size in fluid flow in cancerous tissues [10]. They extended their work by adding solute transport equation and investigating effect of tumor shape and size in drug delivery [11]. In the above-mentioned work, the tissue is modeled as porous media based on Darcy's law and neglects the fluid friction effect.

The momentum equation used in solid tumor modeling in these studies is Darcy's law, but using Darcy's law has some limitations. This model is used only for Newtonian fluid and low velocity flows. Also, in this model the friction within fluid and fluid and solid phase is neglected. In human tissue, all assumptions except neglecting fluid friction are applicable [12].

In the previous works of our group [13, 14], a mathematical model was developed that simultaneously couples interstitial fluid flow with convective noncontinuous blood flow through vessels by considering a remodeling network based on hemodynamic and metabolic stimuli. The effect of vasculature induced by tumor angiogenesis on interstitial pressure is also investigated statistically [15, 16]. In the present work, the mathematical model presented in our previous work is further developed to investigate the effect of fluid friction on solid tumor simulation. In this work, the computational fluid dynamic (CFD) model in our previous studies is developed by considering two models of momentum equation for porous media: Darcy and Brinkman. Three shapes of elliptic tumor are considered in this study: oblate (elongated in *x* direction), circular, and prolate (elongated in *y* direction). The effect of neglecting friction is investigated by comparison of the new model results with the previous one.

## 2. Methods

The mathematical model includes three steps: the modeling of vasculature formation by sprouting angiogenesis, fluid flow in interstitial space, and blood flow through vasculature.

### 2.1. Mathematical Domain

**Figure 1:**
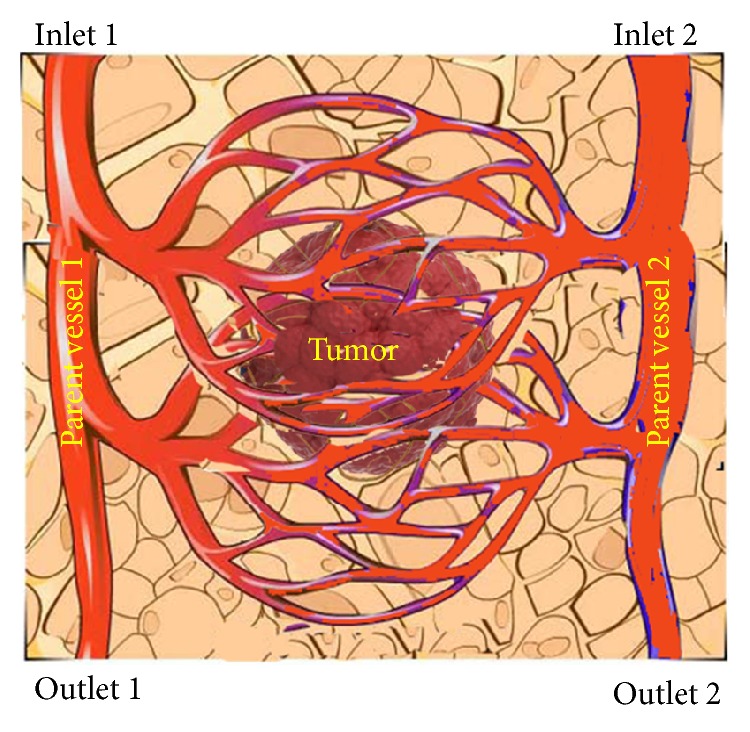
A representation of the solution domain.

Figure 1 shows the 2-dimensional domain considered for the computational studies. The domain is a 4 × 2 cm^2^ rectangle. The two parent vessels are located at both sides of the domain. The elliptic shape is considered for tumor shape. The three different tumor shapes with equal volume are investigated in this study.

### 2.2. Angiogenesis Mathematical Method

The present angiogenesis model is motivated by the tumor-induced angiogenesis model initially proposed by Anderson and Chaplain [17]. The model is a lattice-based model which is derived from the discretization of a 2-dimensional continuous model and used the theory of reinforced random walk motion. The detail of rules for sprouting angiogenesis and algorithm of this method is discussed in both our previous work [18] and Anderson et al. [19].

### 2.3. Intravascular Blood Flow

**Figure 2:**
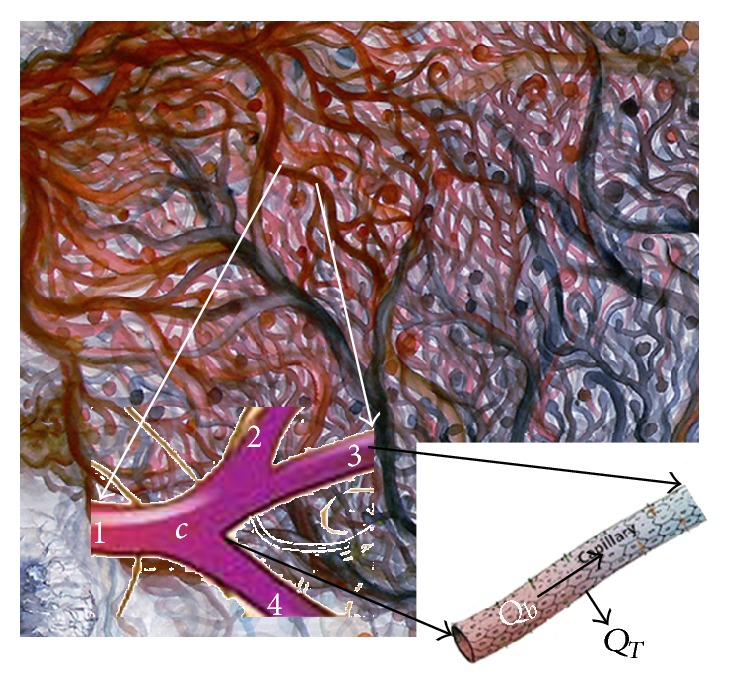
A representation of blood flux at each vascular node.

The blood flow through microvascular network is modeled similar to electrical network. Since blood flow rate through microvascular network corresponds to electric current in electrical network, the balance of blood flow in each interconnecting point like *c* (Figure 2) can be implemented the same as balance of electric current in electrical networks:

(1) $$\begin{array}{c} \overset{N}{\underset{k=1}{\sum }}Q_{c}^{k}=0, \end{array}$$

where *N* is the number of adjacent nodes and *Q*_*c*_^*k*^ is the net blood flow rate:

(2) $$\begin{array}{c} Q_{c}^{k}=Q_{B,c}^{k}-Q_{T,c}^{k}, \end{array}$$

where *Q*_*B*,*c*_^*k*^ is blood flow through each capillary and *Q*_*T*,*c*_^*k*^ is the transvascular flow.

The blood flow through network is modeled by considering noncontinuous behavior of blood and transvascular flow is calculated by Starling's law. The detail of simulation is explained in our previous works [13, 14, 20].

### 2.4. Interstitial Flow

The normal and tumor tissues have characteristics the same as porous media. Interstitial fluid flow in tissues is defined by coupling the fluid flow governing equations: continuity and momentum balance. The mass balance, continuity, equation for steady state incompressible flow in the porous media with source and sink of mass is as follows [1]:

(3) $$\begin{array}{c} \nabla \cdot v_{i}=\phi_{B}-\phi_{L}, \end{array}$$

where *ϕ*_*B*_ is the flow rate from vessel to interstitium or vice versa and *ϕ*_*L*_ is the flow rate from interstitium to lymphatic system. Starling's law represents the fluid flow rate across the microvascular wall [21, 22]:

(4) $$\begin{array}{c} \phi_{B}\left(\;r\;\right)=\frac{L_{P}S}{V}\left(\;P_{B}-P_{i}-\sigma_{T}\left(\;\pi_{B}-\pi_{i}\;\right)\;\right), \end{array}$$

where *S*/*V*, *π*_*B*_, *π*_*i*_, *L*_*P*_, and *σ*_*s*_ are the surface area per unit volume of tissue, the plasma osmotic pressure, the interstitial fluid osmotic pressure, the hydraulic conductivity, and the average osmotic reflection coefficient, respectively.

The lymphatic drainage term is proportional to the pressure difference between the interstitial fluid and the lymphatic vessels [1]:

(5) $$\begin{array}{c} \phi_{L}\left(\;r\;\right)=\frac{L_{PL}S_{L}}{V}\left(\;P_{i}-P_{L}\;\right), \end{array}$$

where *ϕ*_*L*_ is the flow rate into the lymphatic, *L*_*PL*_ *S*_*L*_/*V* is the lymphatic filtration coefficient, and *P*_*L*_ is the hydrostatic pressure of the lymphatic.

**Figure 3:**
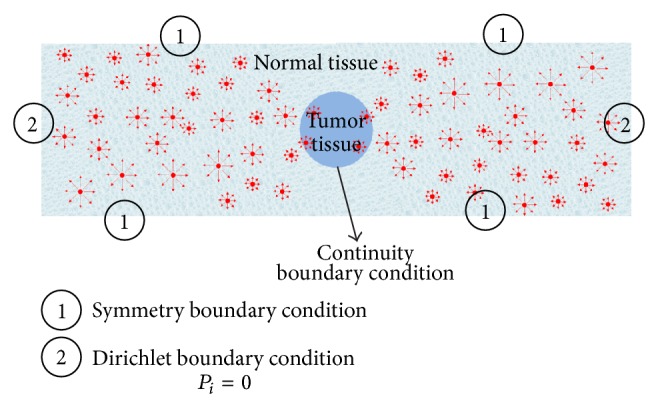
The considered domain and boundary conditions for fluid flow simulation. The 4 parts mentioned in text are shown in this figure. The red circles are capillaries distributed nonuniformly in the domain.

Based on simulation domain shown in Figure 3, the continuity equation is implemented to 4 parts:

- Normal tissue with lymphatic vessel term and capillary term.
- Normal tissue with only lymphatic vessel term.
- Tumor tissue with capillary term.
- Tumor tissue without any capillary or lymphatic vessel term.

The general form of the momentum balance equation in porous media, called Brinkman model, is [2]

(6) ρ∂vi∂t+vi·∇vi=∇·−Pi+μ∇vi+∇viT−2μ3∇·vi−μK+ϕB−ϕLvi+F,

where *K* is the porous medium permeability, *ρ* is the density, *µ* is the viscosity, *v* is the velocity vector, and *F* is the volume forces.

By considering interstitial flow as Newtonian fluid and low velocity for fluid flow and neglecting the friction within the fluid and the fluid and solid phases, (6) in steady state condition changes to Darcy's law:

(7) $$\begin{array}{c} \nabla P_{i}=-\left(\;\frac{\mu }{K}\;\right)v_{i}. \end{array}$$

The *K*/*µ* is defined as interstitium hydraulic conductivity *κ*.

As mentioned in the Introduction, in the interstitium of biological tissues, all Darcy equation assumptions are acceptable except for the friction within the fluid. In this study, the general equation of momentum balance (6) is used.

The boundary conditions applied to the domain for simulation of fluid flow are shown in Figure 3. Further details of the simulation are introduced in our previous work [15].

### 2.5. Model Parameterization

The transport properties for tissues used for interstitial flow modeling are listed in Table 1.

## 3. Results and Discussion

**Figure 4:**
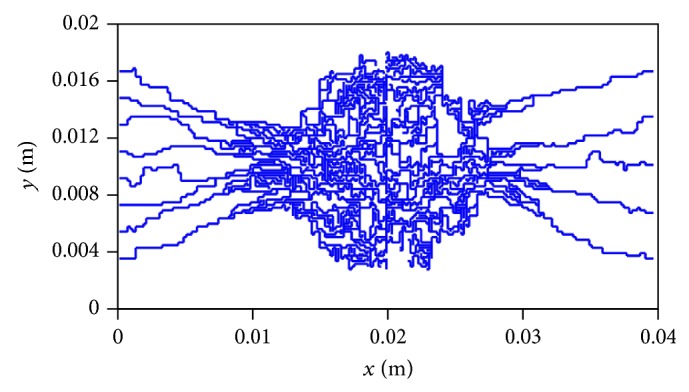
Results of discrete sprouting angiogenesis.

Capillary network obtained by discrete angiogenesis model is presented in Figure 4. The growth of capillary network is simulated by 8 sprouts as initial condition in one parent vessel and 5 sprouts for the other parent vessel. For examining the results for generated network induced by angiogenesis, results obtained by mathematical method are in qualitative agreement with the experimental results carried out in animal corneal models [23, 24] and mathematical model presented by Anderson et al. [17, 19].

**Figure 5:**
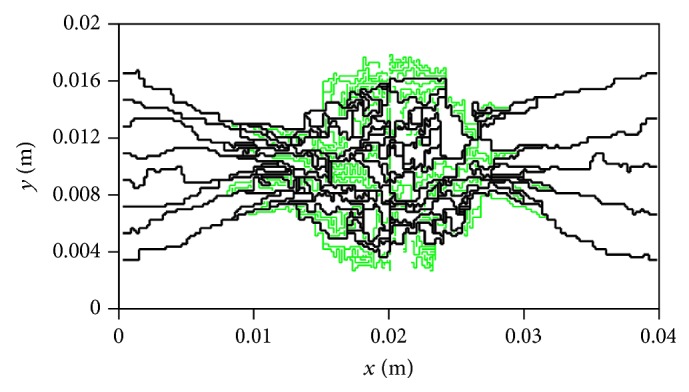
The pruned network used in simulation. The dark lines show the pruned network.

**Figure 6:**
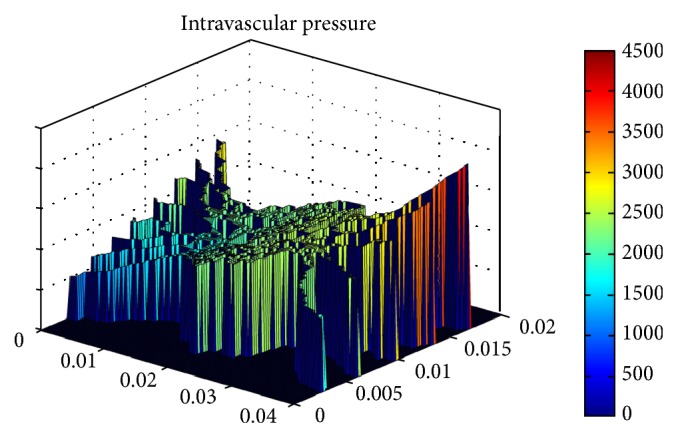
Intravascular blood pressure distribution.

Since the mathematical model which is used for network generation is not a physical model, all vessels do not make a loop in the network. These vessels are removed by a mathematical pruning method before the simulation of blood flow in the capillary network is calculated. The pruned network is shown in Figure 5. The contour of intravascular pressure in capillary network for the second and third approaches is shown in Figure 6.

**Figure 7:**
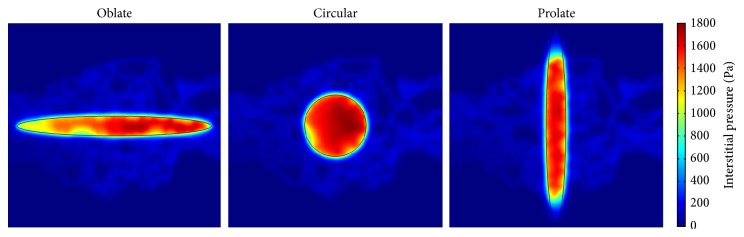
Interstitial pressure distribution in three considered tumor shapes for Darcy model.

**Figure 8:**
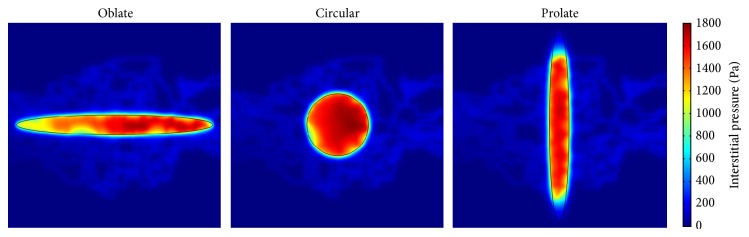
Interstitial pressure distribution in three considered tumor shapes of for Brinkman model.

The interstitial pressure distribution of two considered models and three tumor shapes is shown in Figures 7 and 8, respectively. The interstitial pressure has its maximum value in the tumor region since the vessels in tumor region are leakier than vessels in normal tissue. These effects cause high permeability of vessels and result in more extravascular flow to surrounding tissue based on Starling's law. The lack of lymphatic system in tumor and more flow leakage result in the highest interstitial pressure in the tumor tissue. The highest value of interstitial pressure reported in this study is in a good agreement with [1, 10] and the experimental results of Huber et al. [25]. Results show that the interstitial pressures in both models, Darcy and Brinkman, are very similar. Since interstitial pressure does not depend on fluid friction, the similarity of two models is predictable.

**Figure 9:**
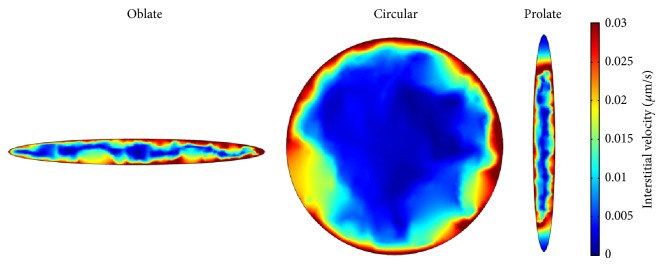
Interstitial velocity distribution in three considered tumor shapes for Darcy model.

**Figure 10:**
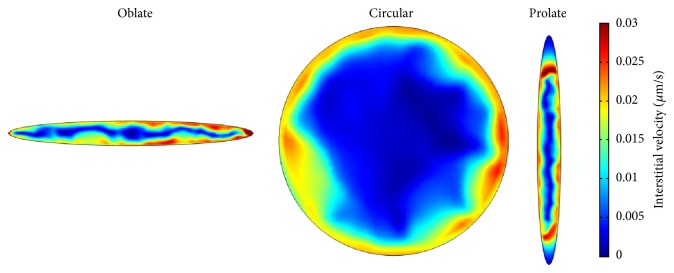
Interstitial velocity distribution in three considered tumor shapes for Brinkman model.

The interstitial velocity distribution at tumor area for two different models is shown in Figures 9 and 10, respectively. The maximum value of interstitial velocity takes place close to the boundary between tumor and normal tissues. Because of low gradient of interstitial pressure in the tumor region, the interstitial velocity value is close to zero. Results show that, in the center of the tumor, the interstitial velocity distributions are similar in two considered models. Far from tumor center, the results of interstitial velocity show difference between two models. Since, near the tumor boundary, the pressure gradient is increased, the velocity is increased as well. Increasing of the velocity in tumor boundary is illustrated in mathematical method [1, 15, 26, 27] and experimental studies [28]. The Darcy model predicts higher velocity than Brinkman model. Considering fluid friction in Brinkman model reduces the effect of pressure gradient and therefore predicts lower velocity than Darcy model.

**Figure 11:**
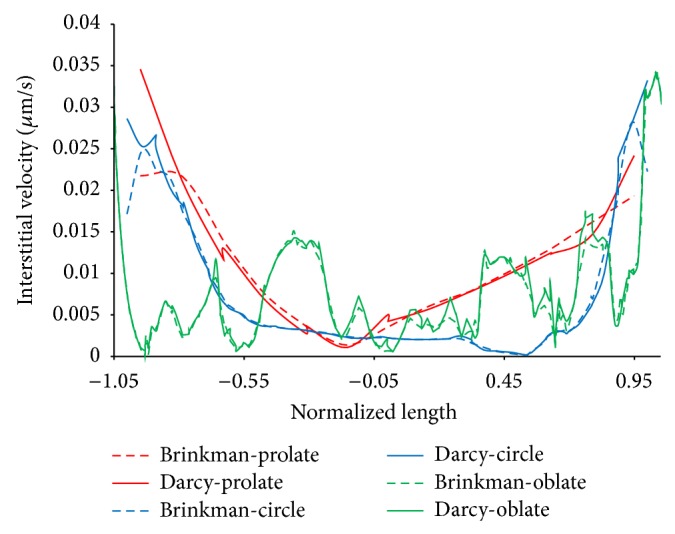
Comparison of interstitial velocity along horizontal line in tumor region for Darcy and Brinkman models.

**Figure 12:**
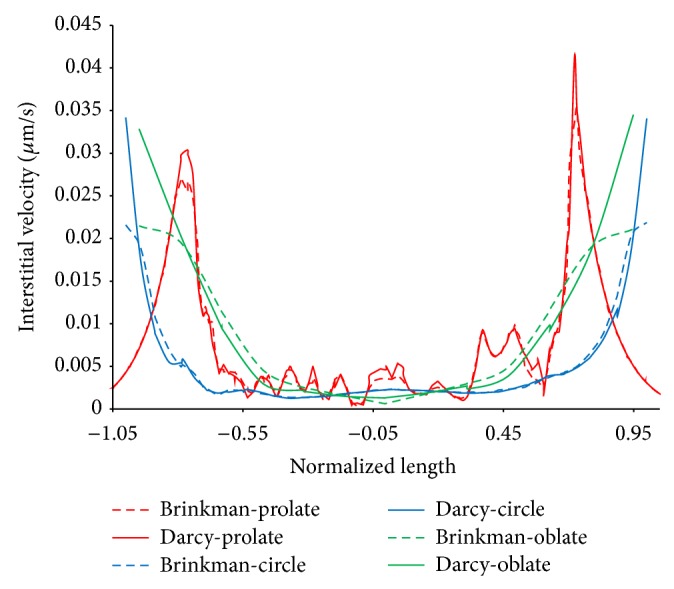
Comparison of interstitial velocity along vertical line in tumor region for Darcy and Brinkman models.

The velocity profiles along horizontal and vertical lines from the tumor center are illustrated in Figures 11 and 12, respectively. Results show that the main differences between two models occurred near the tumor boundary. Prolate shapes show the effect of friction model more significant than other tumor shapes. In prolate type of tumor, the effect of pressure gradient is higher than other shapes of tumor. This effect is investigated in our previous work [11]. With increasing the pressure gradient in tumor region which has important role in the value of velocity, the effect of consideration of fluid friction on interstitial fluid flow is increased.

**Figure 13:**
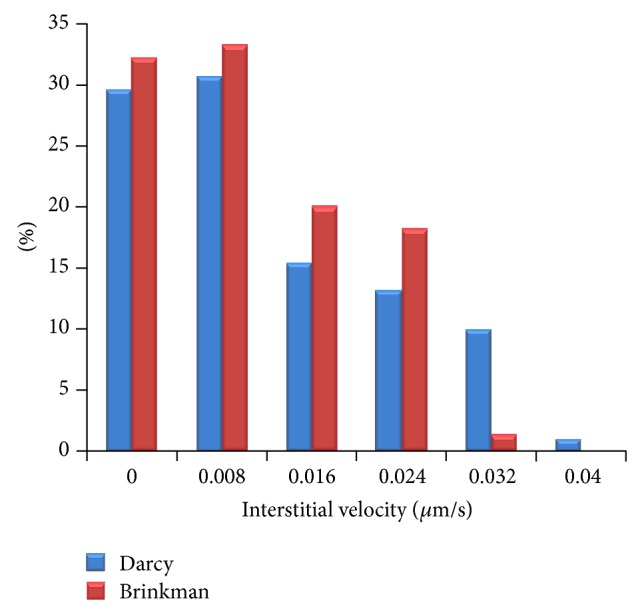
Comparison of interstitial velocity distribution in prolate tumor region for Darcy and Brinkman models.

**Figure 14:**
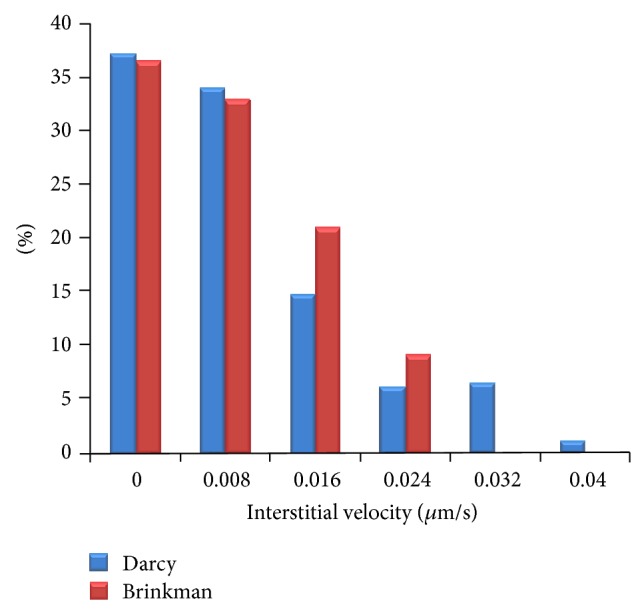
Comparison of interstitial velocity distribution in circle tumor region for Darcy and Brinkman models.

**Figure 15:**
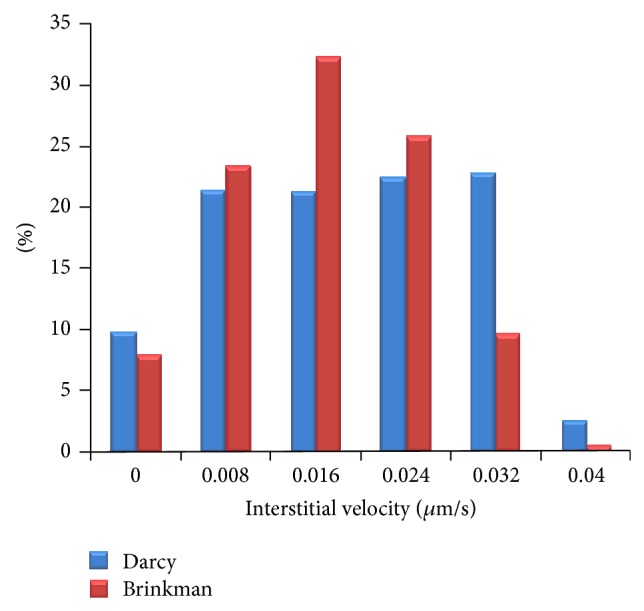
Comparison of interstitial velocity distribution in oblate tumor region for Darcy and Brinkman models.

For showing more clearly the differences between the interstitial fluid flow results of Brinkman and Darcy models, the histogram graphs of interstitial velocity for three shapes of tumors are shown in Figures 13, 14, and 15. In all results, it is clear that the Darcy model has higher values in higher velocity than Brinkman model. In particular, value of high velocity distribution for Brinkman model is negligible or zero.

## 4. Conclusion

In this study, the microvasculature of the tumor induced by tumor factors is modeled based on two well-known models of porous media. The goal of this study is to investigate the fluid friction effects on interstitial flow in different tumor shapes. The results showed that neglecting fluid friction can affect the velocity distribution; however, it has a little effect on interstitial pressure. Generally, the Darcy model predicts higher value for interstitial velocity than Brinkman model. This study shows that friction model is more sensitive in prolate shape than other tumor shapes. The difference in interstitial velocity values leads to a different distribution of drug concentration since the solute transport equation depends on interstitial velocity. Therefore, Brinkman equation is a better assumption for fluid flow modeling in tumors.

## Figures and Tables

**Table 1:** Material properties of normal and tumor tissues used in numerical simulations [11].

Parameter		Baseline value
*L*_*P*_ [m/Pa s]	Normal	2.7 × 10^−12^
	Tumor	21.0 × 10^−12^

*K* [m^2^/Pa s]	Normal	6.41 × 10^−15^
	Tumor	31.05 × 10^−15^

*S*/*V* [m^−1^]	Normal	7000
	Tumor	20000

*π*_*B*_ [Pa]	Normal	2660
	Tumor	2660

*π*_*i*_ [Pa]	Normal	1330
	Tumor	1995

*σ*	Normal	0.91
	Tumor	0.82

*L*_*PL*_ *S*_*L*_/*V* [1/Pa s]	Normal	1.0 × 10^−7^
